# Hand-Fabricated CNT/AgNPs Electrodes using Wax-on-Plastic Platforms for Electro-Immunosensing Application

**DOI:** 10.1038/s41598-019-42644-6

**Published:** 2019-04-16

**Authors:** Sensen Chen, Ahmad Z. Qamar, Narges Asefifeyzabadi, Madison Funneman, Motahareh Taki, Lee Elliot, Mary E. Kinsel, Gary R. Kinsel, Mohtashim H. Shamsi

**Affiliations:** Department of Chemistry & Biochemistry, 1245 Lincoln Dr, Southern Illinois University at Carbondale, Carbondale, IL 62901 USA

## Abstract

Fabrication of inexpensive and flexible electronic and electrochemical sensors is in high demand for a wide range of biochemical and biomedical applications. We explore hand fabrication of CNT modified AgNPs electrodes using wax-on-plastic platforms and their application in electrochemical immunosensing. Wax patterns were printed on polyethylene terephthalate-based substrates to laydown templates for the electrodes. Hand painting was employed to fabricate a silver conductive layer using AgNPs ink applied in the hydrophilic regions of the substrate surrounded by wax. CNT was drop cast on top of the working electrodes to improve their electrochemical signal. The device layers were characterized by scanning electron microscopy. The electrochemical performance of the hand fabricated AgNPs and CNT/AgNPs electrodes was tested using cyclic voltammetry, differential pulse voltammetry, and amperometry. The electrochemical response of CNT/AgNPs electrodes was relatively faster, higher, and more selective than unmodified AgNPs sensing electrodes. Finally, the hand-painted CNT/AgNPs electrodes were applied to detect carcinoembryonic antigen (CEA) by measuring the end-product of immunoassay performed on magnetic particles. The detection limit for CEA was found to be 0.46 ng/mL.

## Introduction

Chemical and bio(chemical) sensing on chips integrated with diverse detection tools are currently hot areas of research^1^. Reports are growing rapidly on the development of novel procedures to fabricate sensor chips and microfluidic platforms^2–4^. Therefore, integration of electronic/electrochemical sensors into flexible devices are in high demand mainly as geared toward point-of-care and wearable sensors^5,6^. For almost a decade, printing methods (i.e. screen, wax, and inkjet) have drawn the attention of the biosensing community as a means to develop inexpensive and simple fabrication procedures^7–18^. Screen-printing has been popular for electrochemical biosensing because it produces reliable electrode surfaces owing to large metal particulates in the ink^19,20^. However, screen-printing requires delicate and thin screens that are tedious to manufacture. Inkjet printing of metallic ink has been widely used for high throughput, instantaneous patterning but it is sensitive to the rheological properties of ink and prone to frequent nozzle clogging^21,22^. These potential problems might be costly in resource deprived settings. Wax printing has emerged as a means to fabricate robust hydrophobic patterns for electrochemical devices on paper substrates^23–25^. Although paper substrates are inexpensive they lack tear toughness that might be required under certain environmental conditions.

Here, we introduce a simpler method of fabrication that combines wax patterning, hand-painting, and drop casting to fabricate sensitive electrochemical sensors on a plastic substrate. Plastic substrates are as inexpensive as paper-based substrates (i.e. $0.50 per letter-sized sheet), but they have high tear toughness which is critical for greater shelf-life. The fabrication strategy used here can be used to construct a variety of electrodes using various materials by avoiding the problems of inkjet printing and screen-printing while maintaining low-cost and robust response. In contrast to paper-based fabrication, wax patterns on plastic substrates do not require heating to create stable hydrophobic barriers between hydrophilic islands^26^. Such thermal stability can benefit fabrication procedures where high temperatures are involved in subsequent steps.

The fabrication procedure used here involved modified polyethylene terephthalate as a substrate, a solid wax printer to create hydrophobic barriers and insulator layers, hand-painting of silver nanoparticles (AgNPs) to lay down a conductive layer, and drop casting of carbon nanotubes (CNT) to enhance the sensitivity of the sensing electrodes. The layers of the electrochemical sensor were characterized by scanning electron microscopy (SEM) to study the integrity of the sensor interfaces. The electrochemical performance of the AgNPs and CNT/AgNPs electrodes were analyzed by cyclic voltammetry (CV) and differential pulse voltammetry (DPV). As a proof of concept, the CNT/AgNPs electrodes were applied for the amperometric detection of carcinoembryonic antigen (CEA), a cancer biomarker, by monitoring an electroactive product released from a magnetic-bead based immunoassay. The immunoassay sensitivity for the CEA was found to be comparable to that of ceramic-based and paper-based screen-printed devices^25^, but the protocol developed here offers a simpler way to FABRICATE robust sensors. Moreover, electrodes comprising CNT-AgNPS mixtures and nanocomposites in absence and presence of polymer additives have been previously reported for improved signaling^27–29^, the results of this work show that the improvement in electrochemical performance can be achieved by simple drop casting of CNT on the AgNPs working electrodes.

## Experimental

### Reagents and equipment

Silver nanoparticles ink (30 wt%, ≤45 μΩ-cm, 28 cP) were purchased from Sigma-Aldrich (Milwaukee, WI). Multiwalled carbon nanotubes (2 g/L, 3 cP) were purchased from NanoLab (Waltham, MA). Carcinoembryonic antigen (CEA), biotinylated primary antibody (Ab_1_), secondary antibody conjugated with horseradish peroxidase (Ab_2_-HRP), and angiogenin were obtained from Fitzgerald Industries International (Acton, MA). Bovine serum albumin solution (30 ± 2%, BSA) and phosphate buffered saline buffer (pH 7.4, 10× concentrate) were purchased from Sigma-Aldrich (Milwaukee, WI). Magnetic beads coated with streptavidin (Dynabeads M-280, dia. 2.8 μm) were purchased from Fisher Scientific. The magnetic beads were then coated with primary antibody (Ab_1_) using the manufacturer described protocol. All solutions were made in PBS buffer (pH 7.4). The Novelle substrate (modified polyethylene terephthalate, PET) was obtained from Novacentrix (Austin, Texas). The wax printer (Xerox ColorQube 8580) was purchased from Xerox Inc. The CNT screen-printed electrodes were purchased from Dropsens (Metrohm, USA). Potentiostat, The CHI 660E, was purchased from CHI Instruments (Austin, Texas) and used for all electrochemical measurements by coupling a commercial adapter (Product of Dropsens) obtained from Metrohm (USA). All wax patterns were designed by using Inkscape (Version 0.91), which is a vector designing software and available free online. Fetal bovine serum (FBS) was kindly provided by Professor Keith Gagnon in the Department of Chemistry and Biochemistry, Southern Illinois University Carbondale. FBS was used after 1:1 dilution in PBS buffer and filtering through a 2 micron filter.

### Fabrication of Electrodes

Sensor patterns were designed using Inkscape (Version 0.91) software. Each sensor chip comprised three-electrodes (working, counter, and reference) with wires and contact pads. The sensor patterns were printed on PET-based substrates (4 *inch* × 6 *inch*) using a Xerox ColorQube 8580 printer. There were 12 sensor chips patterned on a sheet and each sensor chip had dimensions of 2.5 cm in length and 1.0 cm in width. The wax patterns have 60 μm channels and 120 μm line resolutions on these plastic substrates which are stable up to 160 °C^26^. The working electrodes were printed in a circular shape having 1.0–3.0 mm diameter. The areas of the reference and counter electrodes were constant, i.e. 0.049 cm^2^ and 0.238 cm^2^, respectively. The electrode wires were 1.8 cm long and 0.15 cm wide. After the pattern fabrication, a conductive layer of silver nanoparticles was hand-painted in the hydrophilic islands using a paint brush. After air drying for 30 min, any residual silver nanoparticles on top of the hydrophobic wax layer were washed from the sensor with DI water. Next, the conductive patterns were sintered on a hot plate at 120 °C for 15 min. The conducting wires were then covered by printing a wax layer on top of the wires. Finally, a 4 µL aliquot of CNTs was dropped over the central working electrode and allowed to air dry for 1 h followed by sintering at 120 °C.

### Characterization of Electrodes

Laser desorption ionization mass spectrometry (LDI MS) was performed to analyze formulation of the AgNPs ink. A 0.5 μL aliquot of the ink was deposited onto a stainless-steel target and allowed to form a thin film after air drying. Then, the target was inserted into the ion source of a Bruker Daltonics MicroflexRL time-of-flight mass spectrometer with a nitrogen laser operating at 337 nm. The LDI mass spectrum was acquired in linear, positive ion mode. One-hundred laser shots were averaged to generate the mass spectrum. The mass spectrum was internally calibrated using the observed silver cluster ions. The surface structures and properties of the device interfaces were acquired using electron microscopy. SEM was performed using a Quanta 450 FEG (FEI) microscope located in the Image Center facility at SIUC. Elemental composition of the surfaces was obtained using EDX mode in SEM.

### Electrochemical Performance and Optimization

The electrochemical performance of the hand-painted sensors was tested by performing the DPV and amperometry in the presence of electroactive H_2_O_2_-TMB (2 + 5 mM) mixture in PBS buffer (pH 7.4). The electroactive H_2_O_2_-TMB mixture has been used as a substrate in enzyme catalyzed immunoassays^3^. The factors that were studied include the effects of (a) CNT modification, (b) pH, and (c) fabrication batch. Amperometry was used to optimize parameters and to detect the immunoassay product. The following parameters were optimized: (a) catalytic reaction time, (b) sensitivity of the sensor, and (c) size of the working electrodes. These amperometric tests were performed for the catalytic reaction between Ab_2_-HRP (1 µg/mL) and H_2_O_2_-TMB (2 + 5 mM) at 8 mV for 50 s.

### CEA Immunoassay and Amperometric Detection

An ELISA type immunoassay was performed to detect CEA using supramagnetic particles. The immunoassay steps are as followed: (1) a 50 µL aliquot of the Ab_1_-beads suspension was washed with 1 mL of 0.1 M PBS followed by the separation of Ab_1_-beads from the supernatant by placing a magnet underneath, (2) a 1 mL aliquot of CEA biomarker was added into the Ab_1_-beads and allowed to incubate for 1 h at room temperature on a shaker followed by five washing steps, (3) after the separation of ‘CEA/Ab_1_-beads’, 1 µg/mL of Ab_2_-HRP conjugate was added and incubated for 2 h while shaking, as previously reported^30^, followed by five washing steps and separation of the immunoassay complex, i.e. Ab_2_-HRP/CEA/Ab_1_-beads, (4) the enzyme catalyzed reaction was carried out by adding 1 mL of the H_2_O_2_-TMB substrate in the immunoassay complex and allowed to incubate for 5 min at 37 °C, (5) finally, the soluble electroactive product ‘TMB DI’ was separated from the immunoassay complex and placed on to a CNT modified hand-painted sensor to record the amperometric current.

As a negative control, PBS buffer (1 mL) was used in place of the antigen CEA. Angiogenin (1 µg/mL) was used as a positive control. Angiogenin is a protein that expresses in human colorectal cancer cells and can indicate the degree of tumor progression^31^. The immunoassay performance was also studied in the presence of a complex matrix made of fetal bovine serum (FBS). To compare the performance of the hand-fabricated CNT/AgNPs sensors with commercial screen-printed sensors, the amperometric measurements were made for CEA immunoassay on CNT based commercial screen-printed electrodes.

## Results and Discussion

**Figure 1:**
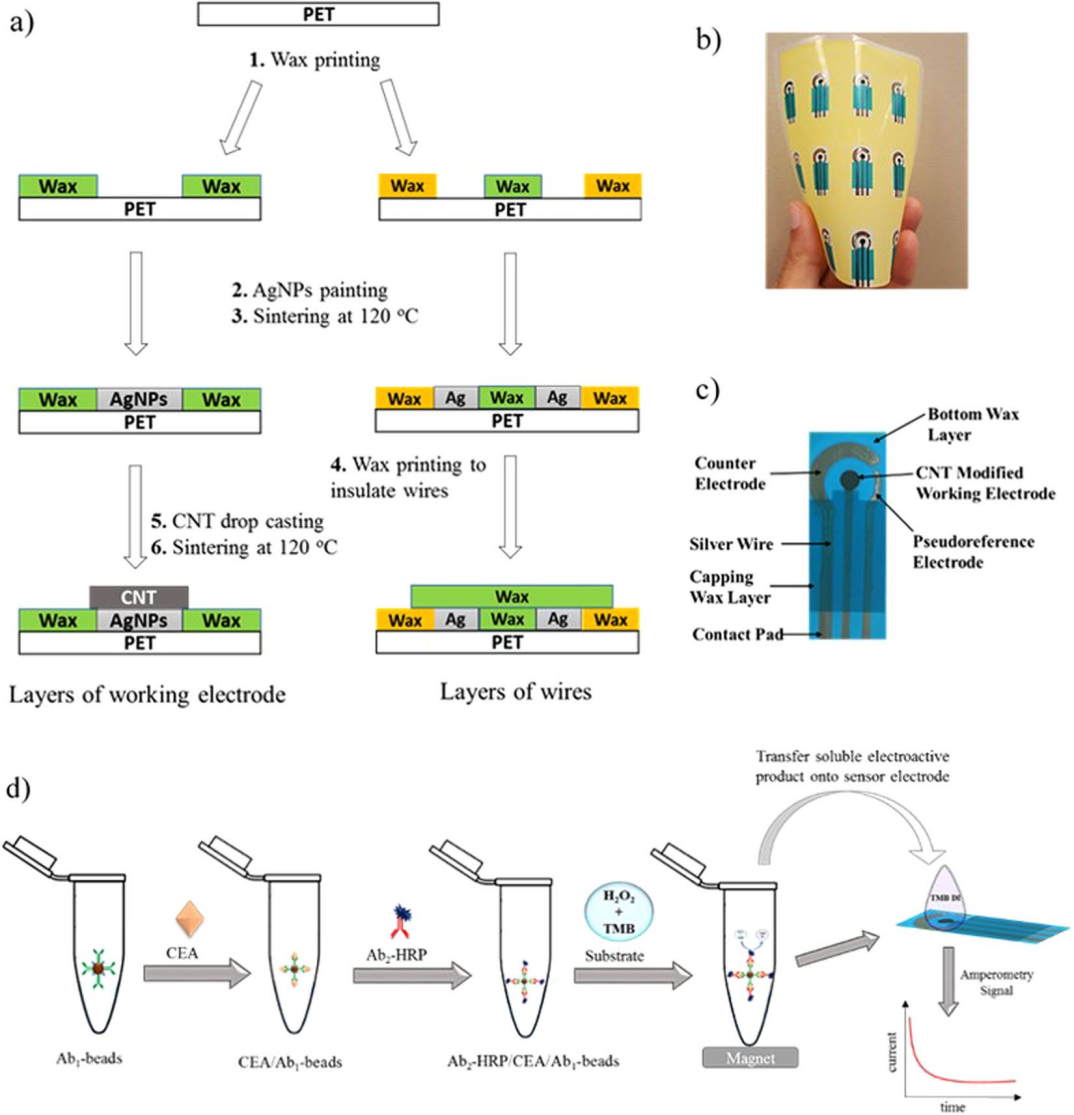
(**a**) Fabrication steps and layering of CNT/AgNPs hand-painted electrochemical sensors. Fabrication steps 1–6 were performed in sequence. In step 4, a wax layer was printed selectively to insulate wires, while in steps 5 and 6, CNT was drop cast on the working electrode followed by sintering. (**b**) Image shows the flexibility of the 12 hand-painted CNT/AgNPs electrochemical sensors on a wax-on-plastic platform. (**c**) Image of a hand-painted CNT/AgNPs electrochemical sensor. (**d**) Illustration describes the sandwich type immunoassay. Ab_1_-beads capture carcinoembryonic antigen (CEA) recognized by the HRP conjugated secondary antibody (Ab_2_-HRP). The addition of H_2_O_2_-TMB substrate produces the electroactive product, TMB DI, which is separated from the Ab_2_-HRP/CEA/Ab1-beads using a permanent magnet and transferred to the hand-painted CNT/AgNPs sensor followed by amperometric current detection.

In this work, we demonstrate the development of wax-on-plastic electrochemical sensors fabricated by hand painting of electrodes on wax patterned flexible plastic. Figure 1 describes the fabrication of hand-painted sensors on wax-on-plastic platforms and illustrates the amperometric detection of CEA using the hand-painted sensors. The device fabrication comprises six key steps: (1) patterning of wax on the plastic substrate using a wax printer to lay down a template of electrodes, (2) hand painting of a silver nanoparticles (AgNPs) layer into the hydrophilic regions of the plastic substrate, (3) sintering of the AgNPs layer to remove solvent, (4) insulating electrode wires by a second wax printing step, (5) drop casting of carbon nanotubes (CNT) on top of the AgNPs working electrodes, and (6) sintering of the CNT layer at 120 °C. Although, composites made of AgNPs and CNT are known for patterning conductive films and electrodes^32,33^, this is the first example of hand-based fabrication of AgNPs followed by drop-casting of the CNT using a wax-on-plastic platform. Moreover, the wax-on-plastic platform is unique because the hydrophobic wax layer on the PET substrate stays stable even above  the melting temperature of wax, as reported previously^26^.

**Figure 2:**
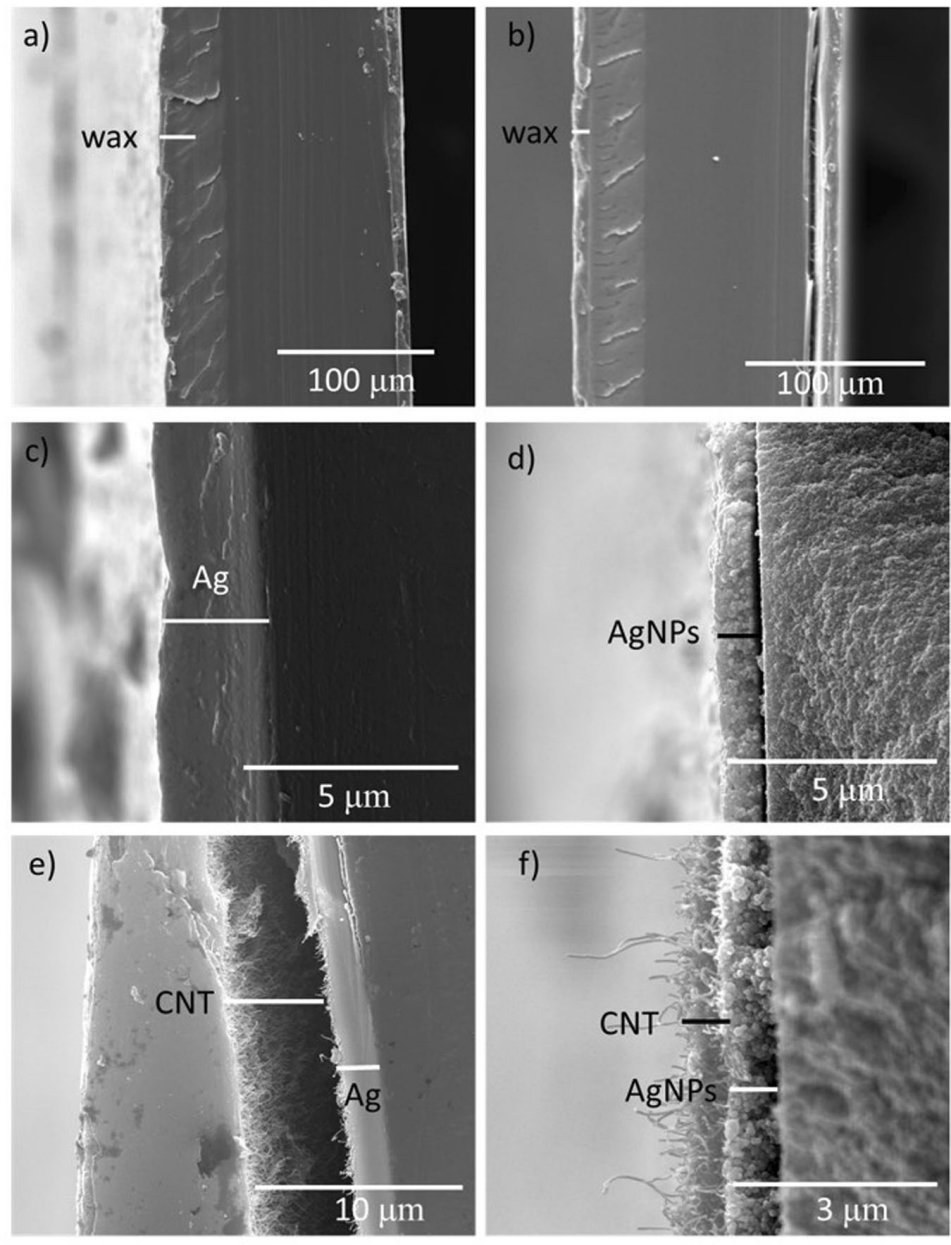
SEM characterizations shows the cross-section views of the device layers before and after sintering at 120 °C for 20 min. The layers are marked by solid white and black lines. Printed wax layer on the multilayered PET substrate before heating (**a**) and after heating (**b**). Hand-painted layer of AgNPs ink before sintering (**c**) and after sintering (**d**). Drop-cast layer of CNT on top of the hand-painted AgNPs layer before sintering (**e**) and after sintering (**f**).

The sintering procedures at 120 °C for 20 min in steps 3 & 6 of the fabrication procedure removed solvent and volatile additives from the AgNPs and CNT leading to coalescence of the nanoparticles. The sintering step was critical to optimize the conductivity of the AgNPs and CNT as prescribed in the product information. The fidelity of the device layers and their thicknesses before and after the sintering procedure was characterized by SEM. Figure 2(a) shows that the wax layer before sintering was found to be 20 ± 5 μm thick and well-adhered to 140 μm thick PET substrate. The effect of heating on the wax layer (Fig. 2(b)) was consistent with our previously reported results^26^. Specifically, while the wax layer appeared stable at this temperature the thickness of the layer decreased by up to 10 μm and may have increased in width in the xy plane a similar amount. The variation in the pattern in the xy plane at this length scale is not a major concern in our fabrication due to significantly larger dimensions in the millimeter scale. Figure 2(c) shows the thickness of the hand-painted silver layer before the sintering step. The Ag ink layer was analyzed in the electrode and wire regions of at least six samples where the thickness before sintering was measured to be 3.10 ± 0.24 µm. As expected, sintering removed the solvent and capping agents that allowed the AgNPs to coalesce to form a layer with average thickness of 0.86 ± 0.14 µm, Fig. 2(d). Drop casting of 4 µL of the CNT produces a 10.26 ± 2.16 µm thick layer before sintering marked on top of the silver layer in Fig. 2(e). Sintering adhered the CNT layer on the AgNPs with an average thickness of 0.46 ± 0.15 µm (Fig. 2(f)). The fidelity of the hand-fabricated electrodes for consistent electrochemical response is an obvious concern and the issue was addressed by testing the electrochemical response of various batches of the electrodes.

The amperometric test was performed to study the effect of manual painting on the electrochemical performance of the CNT/AgNPs electrodes using an electroactive mixture of H_2_O_2_-TMB. Figure S1 in the supporting information shows the amperometric current responses of four batches. The statistical test for four batches involving single factor ANOVA confirmed that the difference between the batches is not significant.

The electrochemical performance of the hand-painted AgNPs and CNT/AgNPs electrodes were first tested for fast electron kinetics by monitoring the CV response of Fe(CN)_6_^3−/4−^ (Fig. S2 in supplementary information). Theoretically, fast electron transfer kinetics, also known as a reversible process or Nernstian behavior, should show a peak separation of ~59 mV between the oxidation and reduction peaks per electron transfer. The AgNPs electrodes were found to be extremely inactive, which is evident from the peak separation of ~500 mV (see Fig. S2a). However, CNT modification increased the current response and improved the electron kinetics by reducing the peak separation to half, i.e. ~240 mV (see Fig. S2b). In a control experiment, CNT was drop cast on a cleaned indium tin oxide (ITO) surface and tested for reversible response of Fe(CN)_6_^3−/4−^. Figure S3 (supplementary information) shows the CV scans of CNT/ITO electrodes measured for scan rates 5 to 50 mV/s, which verified the reversible electron kinetics with a peak separation of ~55 mV per electron transfer along with a linear current increase with the square root of the scan rates and without significant change in peak positions–both behaviors are the signs of fast electron kinetics. It was hypothesized that the slow kinetics of the hand-painted AgNPs and CNT/AgNPs electrodes was due to the presence of additives in the AgNPs ink. Therefore, the AgNPs ink was analyzed by LDI MS to identify the additives. The LDI MS spectrum (Fig. S4 in the supporting information) showed a pattern of polymer-like ion signals in the mass spectrum suggesting the presence of a silver attached polymer having a repeat unit of 117.6 Da. The polymer may have been added as a binder or a capping agent. However, this information was not found in the manufacturer’s product description. According to the product information, the AgNPs were dispersed in ethylene glycol, however no ethylene glycol ion signal was observed in the mass spectrum. From these experiments, it was concluded that a significant polymer content is present in the AgNPs ink, which may be the main hindrance in achieving fast electrode kinetics in the hand-fabricated CNT/AgNPs electrodes.

**Figure 3:**
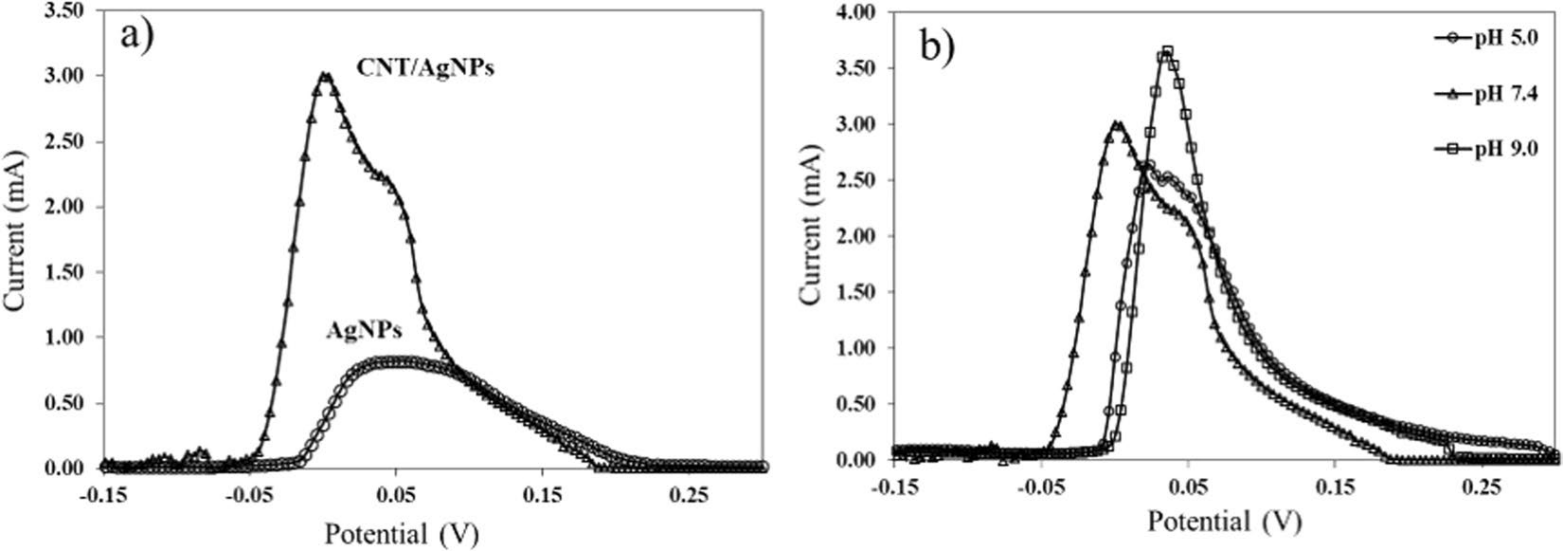
(**a**) DPV curves for H_2_O_2_-TMB (2 + 5 mM) mixture in PBS buffer (pH 7.4) using hand-painted AgNPs electrodes (◦) and CNT/AgNPs electrodes (∆). (**b**) DPV curves for H_2_O_2_-TMB using CNT/AgNPs electrodes in PBS buffer at pH 5.0 (◦), pH 7.4 (∆), and pH 9.0 (□). Diameter of the working electrode is 2.5 mm.

The electrochemical performance of the AgNPs and CNT/AgNPs electrodes were characterized by DPV, which is a sensitive electrochemical technique that results in a current peak at the formal potential of a redox couple^34^. Figure 3(a) shows the DPV response of the H_2_O_2_-TMB recorded on AgNPs and CNT/AgNPs hand-fabricated electrodes. For the AgNPs electrode, a broad peak was observed between 30–75 mV with a maximum peak current of 0.8 mA. In contrast, the CNT addition to the AgNPs enhances the current response by almost a factor of four leading to a maximum peak current of 3 mA. It is noteworthy that the electrochemical signal amplification by CNT addition has been previously attributed to a higher surface area offered by the multiwalled carbon nanotubes^35^. Moreover, a shoulder on the right side of the peak was also observed, which indicates that the CNT/AgNPs electrode can be somewhat selective towards the TMB and H_2_O_2_ oxidation processes due to relatively active surface of the CNT as confirmed by the cyclic voltametric measurements (Fig. S2).

Further experiments to optimize the immunoassay parameters were performed using CNT/AgNPs electrodes. Figure 3(b) shows the effect of pH on the current response of CNT/AgNPs electrodes using the DPV technique. A trend of increasing peak current from pH 5–9 and a shift in potential is observed, where the highest peak current of 3.5 mA was observed at a 40 mV potential for pH 9.0. Such behavior is generally attributed to systems that involve consumption and production of H^+^, which affects the overpotentials and current intensities^36^.

**Figure 4:**
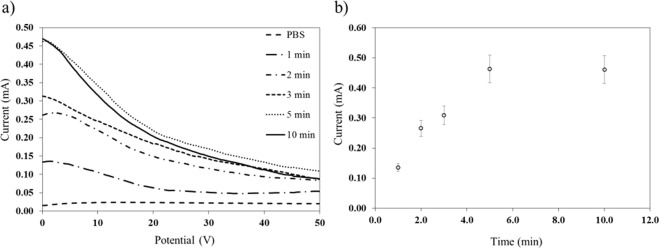
(**a**) Representative amperometric curves for the reaction between 1 μg/mL Ab_2_-HRP and H_2_O_2_-TMB (2 mM + 5 mM) substrate at different incubation periods using CNT/AgNPs electrodes. (**b**) Plot of current versus incubation time obtained from the amperometric curves. Error bars represent standard deviation for n = 3. Amperometry was performed at pH 7.4, diameter of working electrodes 2.5 mm, and applied potential 8 mV.

In an enzyme catalyzed immunoassay, signal is typically generated by a chemical species produced as a result of a reaction between an enzyme and a substrate. The immunoassay performed here involves the reaction between Ab_2_-HRP (an enzyme conjugated secondary antibody) and H_2_O_2_-TMB (a substrate). The reaction time was optimized by monitoring the amperometric current of the product using the CNT/AgNPs electrodes. Figure 4(a) shows the optimization of reaction time as measured by amperometry at 8 mV (pH 7.4) over 2.5 mm diameter working electrodes. Figure 4(b) verifies that the current response increases with the increase in time of reaction and levels off after 5 min of incubation and this incubation time was used in further studies.

**Figure 5:**
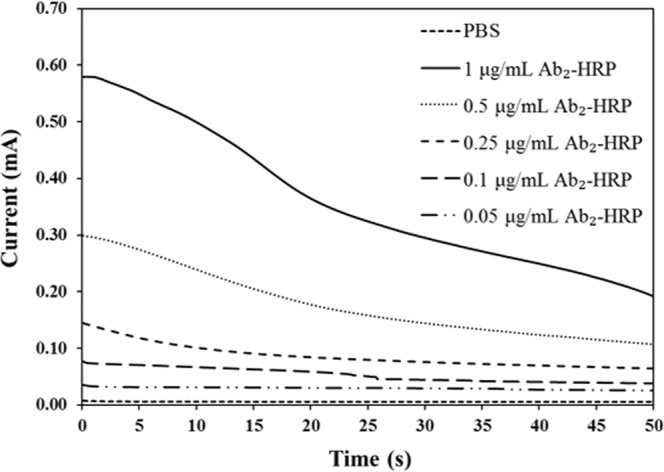
Representative amperometric curves for the reaction between Ab_2_-HRP and H_2_O_2_-TMB at different concentrations of Ab_2_-HRP using 2.5 mm diameter CNT/AgNPs electrodes.

Figure 5 shows the sensitivity of the CNT/AgNPs hand-fabricated electrodes toward the concentration of Ab_2_-HRP. The amperometric curves were obtained for the catalytic reaction between TMB-H_2_O_2_ substrate and 0.05–1.00 μg/mL Ab_2_-HRP. The amperometric current was found to be linearly related to the concentration and the calibration sensitivity increased with the area of the working electrodes (see Fig. S5 in supporting information). The dynamic range covers two orders of magnitude and the increase in the slope of the calibration curves with the area of the working electrodes is consistent with the theoretical model of the Randle-Sevcik expression, i.e. current is directly proportional to the area of working electrode^36^. For the electro-immunosensing application, the 1.0 mm diameter working electrode was selected because of the lowest background current (noise).

**Figure 6:**
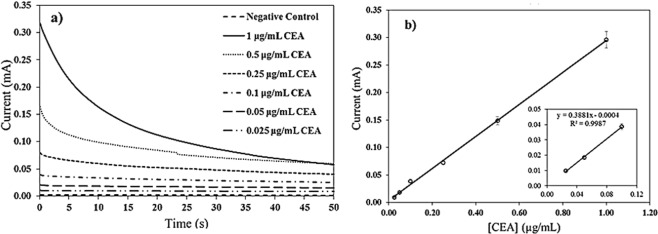
(**a**) Amperometric curves for the detection of CEA using 1.0 mm diameter CNT/AgNPs electrodes. Negative control was 1 mL of PBS buffer. (**b**) Calibration curve for CEA for the CEA concentration range 0.025–1.00 μg/mL. Inset shows the current for 25–100 ng/mL with straight-line equation.

As a final proof of concept, the CNT/AgNPs hand-fabricated electrodes were applied to detect the amperometric current from the carcinoembryonic antigen (source: purified human liver carcinoma >80% purity) immunoassay. As illustrated in Fig. 1, the sandwich type immunoassay was performed on magnetic particle surfaces modified to capture CEA followed by amperometric detection. Specifically, the Ab_1_-modified-bead captures the CEA antigen, which is then recognized by the Ab_2_-HRP (enzyme-secondary antibody conjugate). The subsequent addition of H_2_O_2_-TMB substrate initiates an enzymatic reaction that produces electroactive TMB DI for the amperometric detection. The electroactive product TMB DI is separated from the bead suspension by placing a magnet under the container and a 5 μL drop of the product is placed on a sensing electrode for amperometric measurement. Figure 6(a) shows the amperometric response of the immunoassay as a function of CEA concentration. Figure 6(b) shows a calibration curve for a CEA concentration range of 0.025–1.00 μg/mL. The inset in Fig. 6(b) shows the current values for the concentrations 25–100 ng/mL along with the straight-line equation. The detection limit (LOD) for CEA was calculated using equation LOD = 3 × (standard deviation of blank) divided by the slope of the calibration curve^37^.

As a negative control, 1 mL of a PBS buffer was used that yields a blank current 2.1 ± 0.06 μA. Thus, the calculated LOD for CEA using the CNT/AgNPs hand-painted electrodes was 0.46 ng/mL. This LOD for CEA using the CNT/AgNPS is 10× lower than the clinical cut-off value. In healthy individuals, the normal CEA level is in the range of 3–5 ng/mL in blood^38^. Since commercial screen printed electrodes are widely used for electrochemical immunoassays, it is appropriate to question the utility of the hand-fabricated electrodes given the presence of these commercial electrodes. Therefore, the end product of the CEA immunoassay was analyzed in parallel workflow on the commercial CNT based screen-printed electrodes. Figure S6 shows the amperometric response and calibration curve obtained from the commercial electrodes (Dropsens, 4.0 mm dia.), which lead to a LOD of 0.38 ng/mL for CEA, comparable to the hand-fabricated CNT/AgNPs electrodes. Table 1 compares the results with the recent report on the CEA immunoassay using a paper-based device produced by screen-printing of gold nanoparticles (AuNPs) electrodes. The LOD for the CEA was reported to be 0.33 ng/mL on the paper substrate^25^.

**Table 1:** Comparison of the performances of various electrodes for detecting CEA.

Electrode material	Substrate	Fabrication	Electrode dia.	LOD	Source
AuNPs	Paper	Screen-printed	Not given	0.33 ng/mL	ref.^25^
CNT	Ceramic	Screen-printed	4.0 mm	0.38 ng/mL	Commercial
CNT/AgNPs	Plastic	Hand-painted	1.0 mm	0.46 ng/mL	This work

**Figure 7:**
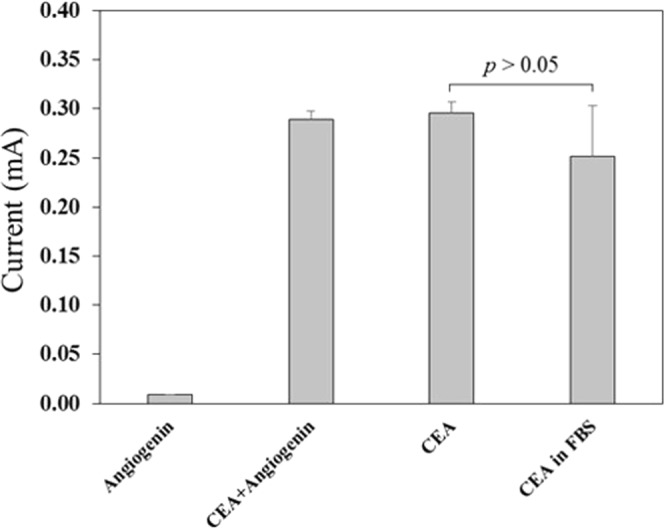
Specificity test. Comparing the current response of 1 μg/mL CEA analyte, positive control (angiogenin), mixture of CEA and angiogenin (1:1), and 1 μg/mL CEA in fetal bovine serum (FBS). Statistical t-Test assuming unequal variances for CEA and CEA in FBS turns out to be *p* > 0.05 for n = 12.

The specificity of the immunoassay system was also tested using a non-specific protein, angiogenin, which signals cancer progression. Figure 7 shows the current response of the angiogenin (positive control), mixture of angiogenin and CEA, and CEA antigen in PBS buffer. Statistically, the effect of the non-specific adsorption of angiogenin was found to be insignificant. The effect of a complex matrix was also tested using fetal bovine serum (FBA) in the antigen medium. This matrix apparently interferes with the immunoassay leading to loss in current response. This behavior may be due to the loss in electroactive product formation, which eventually lowers the current response. It is important to mention that FBS was present in the first step of the immunoassay, (i.e. antigen binding event), while it was washed away in the subsequent steps. This insight suggests that the lower current response in the complex matrix is not because of the inefficiency of the CNT/AgNPs electrodes, but rather due to the nonspecific adsorption of matrix material on the magnetic particles surfaces, which can be rectified using improved washing procedures for the assay.

## Conclusion

In this report, a simplified fabrication of an electrochemical sensor involving wax patterning on plastic, hand painting of AgNPs to lay down a conducting layer, and simple drop casting of CNT to improve electrochemical performance of the sensor is described. The device layers were found to be well adhered to each other and the wax layer can sustain sintering up to a temperature of 120 °C. The fabrication procedure was found to be reliable from batch to batch as tested by amperometric current response. In addition, the electrochemical response of AgNPs electrodes was enhanced by simple drop casting of CNT. As a result of the modification, the CV response was improved by decreasing the peak separation from 500 mV to 240 mV. The DPV response was found to be enhanced by a factor of 4 in current intensity. Amperometrically, the CNT/AgNPs sensors responded linearly to analyte concentrations, area of working electrode, and catalytic product of the CEA immunoassay. The limit of detection for CEA was *ca*. 0.46 ng/mL, which is 10x lower than the clinical cutoff value and comparable to the performance of expensive commercial screen-printed electrodes and recently reported paper-based screen-printed sensors. Considering the advantages of the fabrication approach in this work, the CNT/AgNPs electrodes on wax-on-plastic platforms have real potential for immediate applications, such as straightforward electrochemical assays in resource-limited settings.

## Supplementary information

Supplementary Information

## Data Availability

The datasets generated during and/or analyzed during the current study are available from the corresponding author on reasonable request.
