# Supplementary material for: Hand-Fabricated CNT/AgNPs Electrodes using Wax-on-Plastic Platforms for Electro-Immunosensing Application
Source: Sci Rep. 2019 Apr 16;9:6131. doi: 10.1038/s41598-019-42644-6 (PMC6467877; doi:10.1038/s41598-019-42644-6)
Supplement: Supplementary file 1 — Supplementary Information [file 41598_2019_42644_MOESM1_ESM.docx]

**SUPPLEMENTARY INFORMATION**

**Hand-Fabricated CNT/AgNPs Electrodes using Wax-on-Plastic Platforms for Electro-Immunosensing Application**

Sensen Chen, Ahmad Z. Qamar, Narges Asefifeyzabadi, Madison Funneman, Motahareh Taki, Lee Elliot, Mary E. Kinsel, Gary R. Kinsel, Mohtashim H. Shamsi*

Department of Chemistry & Biochemistry, 1245 Lincoln Dr,

Southern Illinois University at Carbondale, IL 62901, USA

*Email: [mshamsi@siu.edu](mailto:mshamsi@siu.edu)


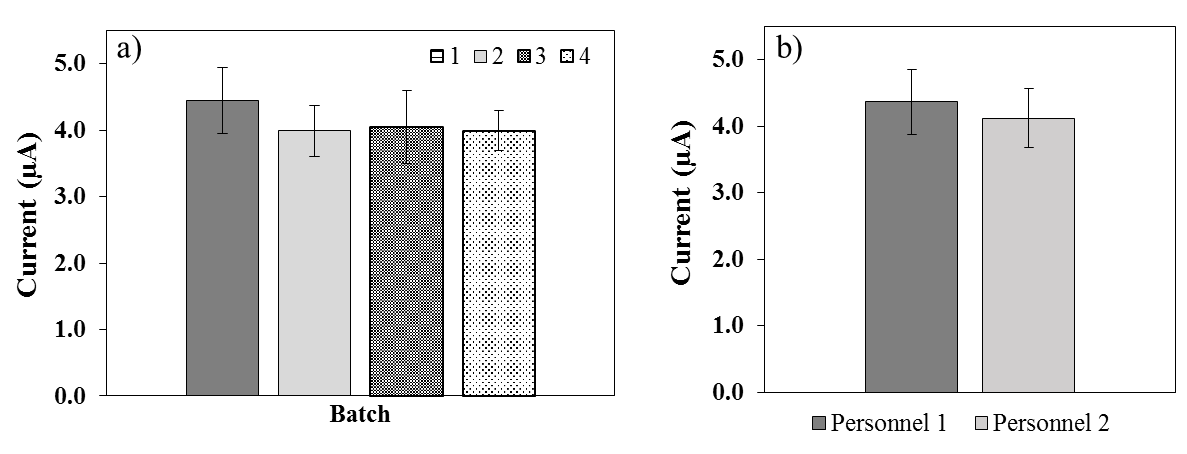
**Figure S1.** Batch effect on the amperometric response of CNT modified hand-painted devices made by painter 1 using H_2_O_2_-TMB mixture. Error bars represent standard deviation with n=4 and *p* > 0.05 at 95% confidence interval, *F_cal_* (0.99) < *F_crit_* (3.49). Amperometry was performed at pH 7.4, diameter of working electrodes 2.5 mm, and applied potential 8 mV.


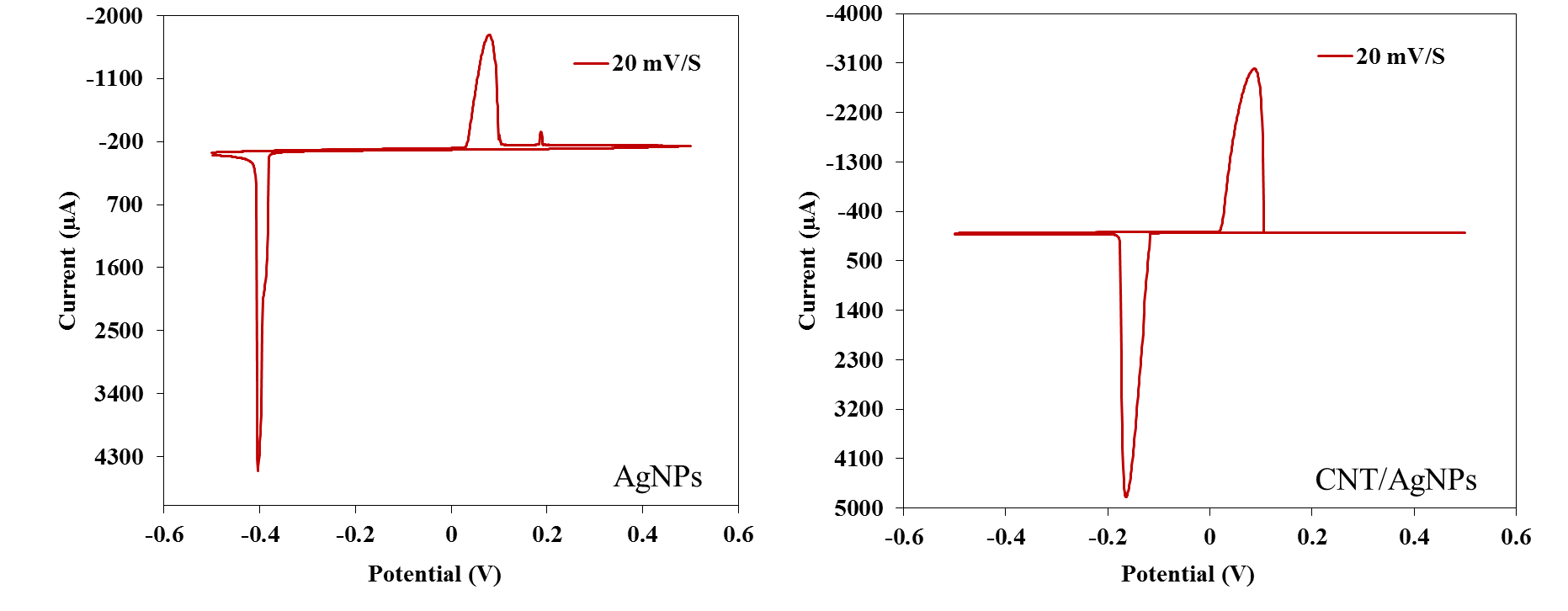


a)

b)

**Figure S2.** Cyclic voltammetric response of 1 mM Fe(CN)_6_^3-^/^4-^ in PBS buffer recorded on (a) AgNPs and (b) CNT/AgNPS hand-painted electrodes at 20 mV/s scan rate.


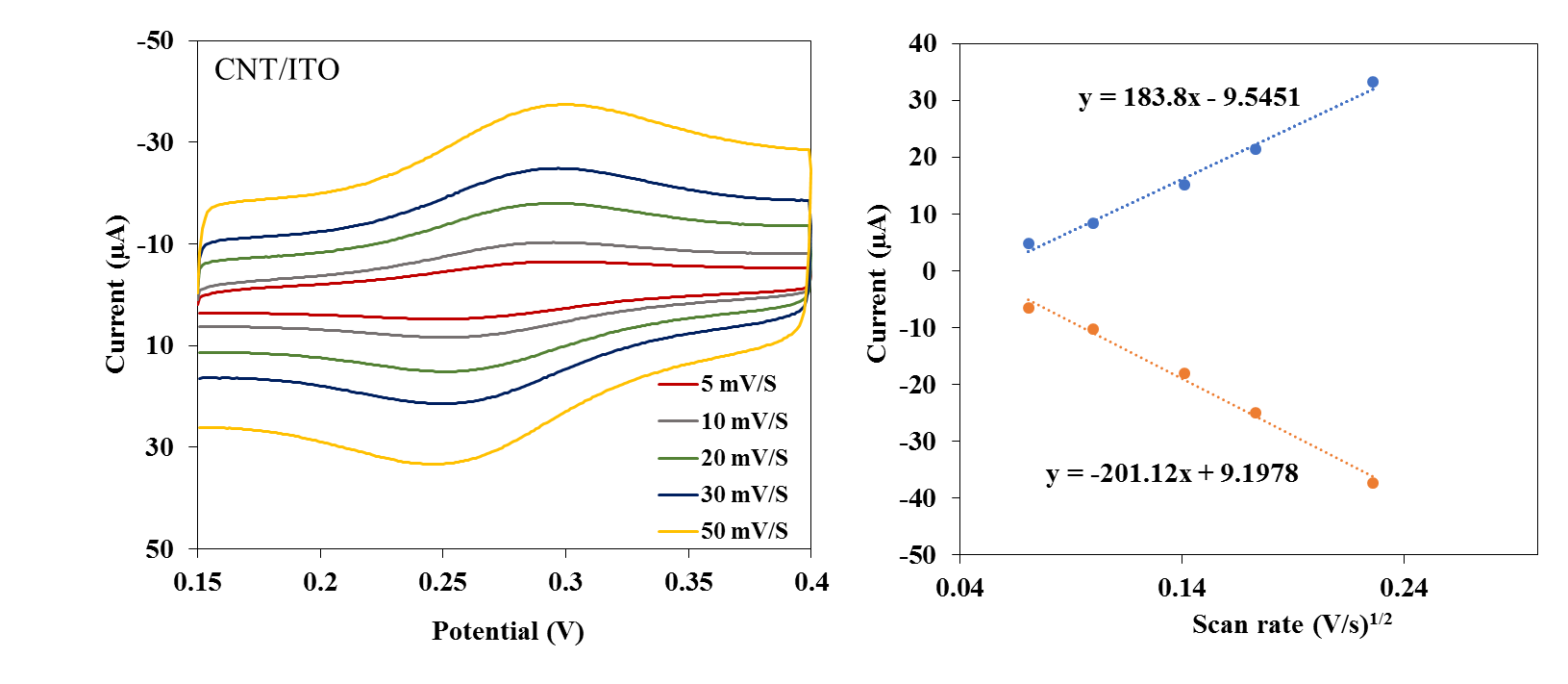


**Figure S3.** Left: Cyclic voltammetric response of 1 mM Fe(CN)_6_^3-^/^4-^ in PBS buffer recorded on CNT/ITO electrodes at 5-50 mV/s scan rate against Ag/AgCl reference. Right: Curves showing linear relationship between oxidation/reduction peak currents and scan rate.

**Figure S4.** Laser desorption ionization mass spectrum of the 30% silver nanoparticles ink.

The LDI mass spectrum in Figure S3 (supporting information) shows silver cluster ion signals appearing at mass-to-charge (m/z) 106.9/108.9 (Ag+), 213.9/215.9/217.9 (Ag2+) and 320.7/322.7/324.7/326.7 (Ag3+) for the two silver isotopes (natural abundance), 107Ag (51.84%) and 109Ag (48.16%). Ion signals are also seen at m/z 273.0/275.0, 390.7/392.7, and 508.3/510.3 indicating the presence of a silver attached polymer having a repeat of 117.6 mass units. This polymer may have been used as a capping agent in the formulation (not mentioned in the product information). Although the AgNPs were dispersed in ethylene glycol, no ethylene glycol ion signal was observed in the mass spectrum. Identification of the polymer used in the silver ink formulation is not within the scope of the work presented here. Nevertheless, the presence of this unknown polymer in the AgNPs formulation (not quantified here) could be responsible for hindering the fast electron transfer kinetics at the electrode interface.

Peak assignments for Ag_2_^+^clusters:

^107^Ag + ^107^Ag 🡪 213.9

^107^Ag + ^108^Ag 🡪 215.9

^109^Ag + ^109^Ag 🡪 217.9

Peak assignments for Ag_3_^+^clusters:

^107^Ag + ^107^Ag + ^107^Ag 🡪 320.7

^107^Ag + ^107^Ag + ^109^Ag 🡪 322.7

^107^Ag + ^109^Ag + ^109^Ag 🡪 324.7

^109^Ag + ^109^Ag + ^109^Ag 🡪 326.7

**
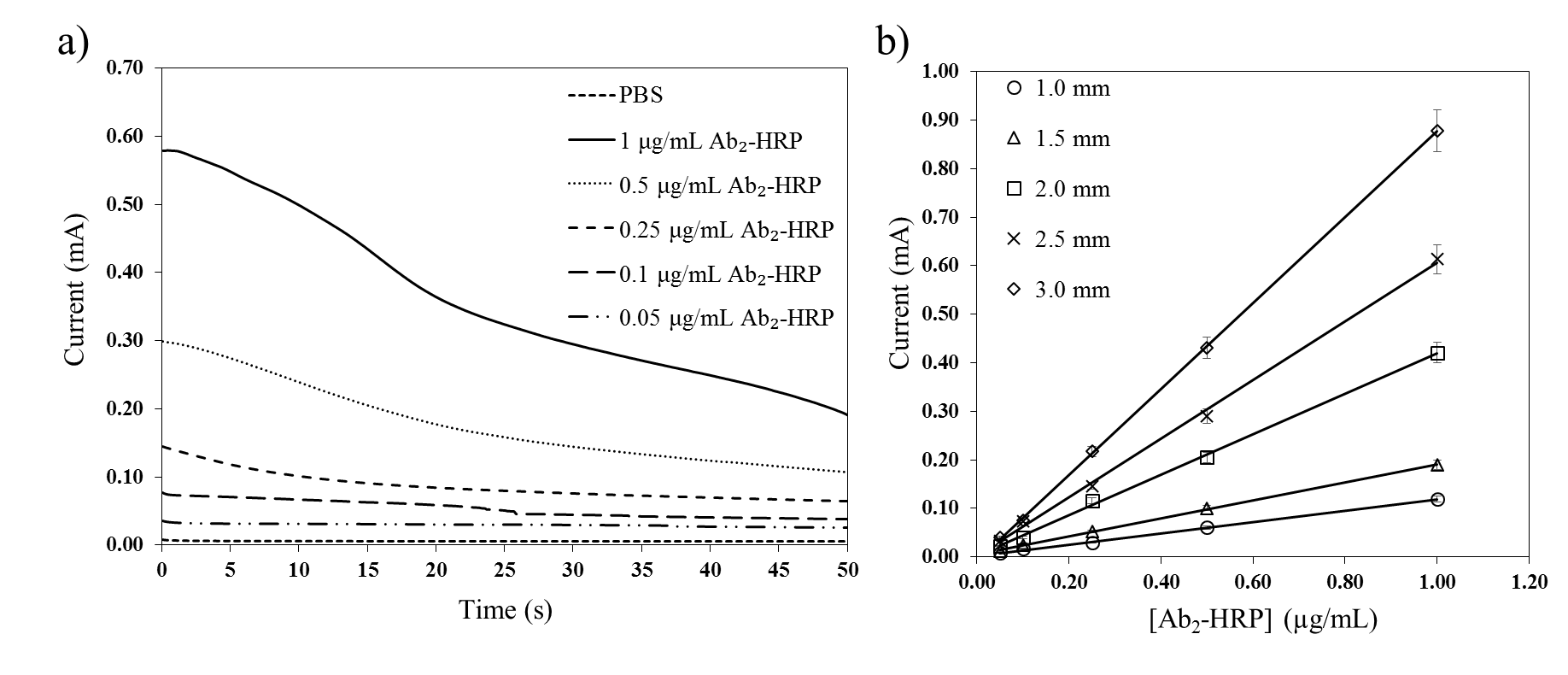
**

**Figure S5**. Calibration curves for [Ab_2_-HRP] as a function of area of working electrodes using CNT/AgNPs electrodes. Amperometric current collected at 1 s was used to plot calibration curves. Error bars represent standard deviation for n=3-5. The reaction conditions include 1 μg/mL Ab_2_-HRP, (2 mM + 5 mM) H_2_O_2_-TMB, 5 min incubation time, pH = 7.4.


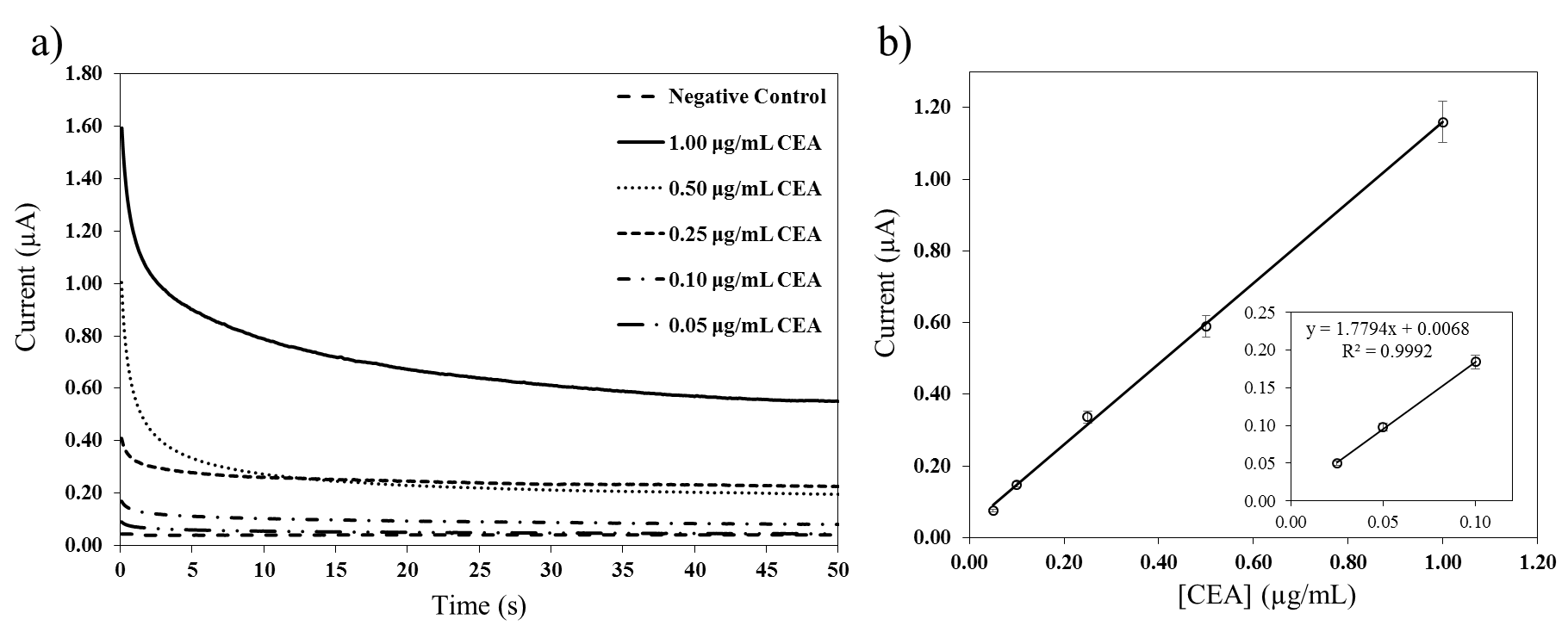


**Figure S6**. a) Amperometric curve for different concentrations of CEA analyzed on CNT screen-printed electrodes. In the negative control, 1 mL of PBS was added in place of CEA. (b) Calibration curve for [CEA] derived from CNT screen-printed devices with the detection limit of 0.38 ng/mL. Error bar represents standard deviation with n=3. Experimental conditions include substrate added at pH 9, 4.0 mm diameter working electrodes, and 220 mV applied potential.

1. K. F. Chan, H. N. Lim, N. Shams, S. Jayabal, A. Pandikumar and N. M. Huang, *Materials Science and Engineering: C*, 2016, **58**, 666-674.
